# Liposomes and Lipid Droplets Display a Reversal of Charge-Induced Hydration Asymmetry

**DOI:** 10.1021/acs.nanolett.3c02653

**Published:** 2023-10-23

**Authors:** Saranya Pullanchery, Nathan Dupertuis, Tereza Roesel, Sylvie Roke

**Affiliations:** † Laboratory for Fundamental BioPhotonics (LBP), Institute of Bioengineering (IBI), School of Engineering (STI), École Polytechnique Fédérale de Lausanne (EPFL), CH-1015 Lausanne, Switzerland; ‡ Institute of Materials Science (IMX), École Polytechnique Fédérale de Lausanne (EPFL), CH-1015 Lausanne, Switzerland; § Lausanne Centre for Ultrafast Science (LACUS), École Polytechnique Fédérale de Lausanne (EPFL), CH-1015 Lausanne, Switzerland

**Keywords:** water, hydrogen bonding, molecular ordering, lipid membrane, intermolecular interactions, second harmonic generation, scattering, charge hydration asymmetry

## Abstract

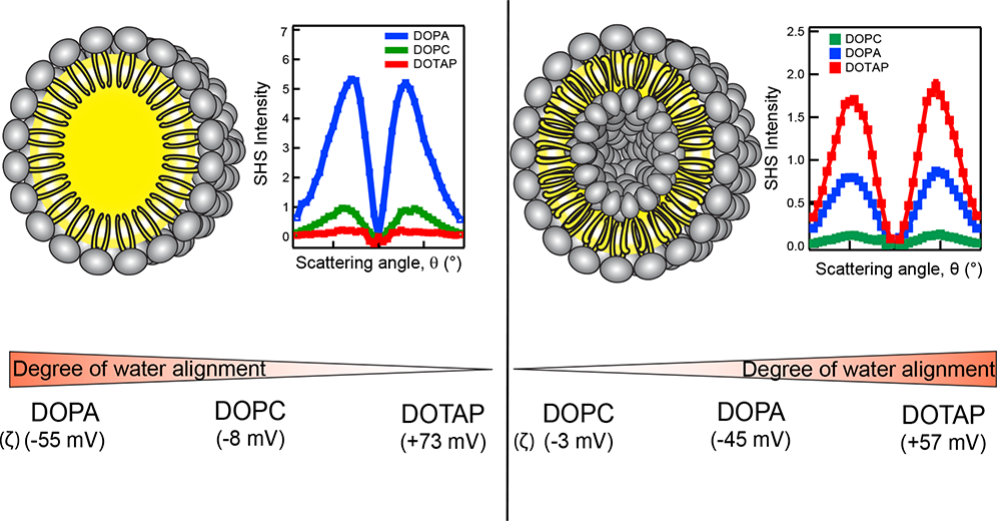

The unique properties of water are critical for life. Water molecules have been reported to hydrate cations and anions asymmetrically in bulk water, being a key element in the balance of biochemical interactions. We show here that this behavior extends to charged lipid nanoscale interfaces. Charge hydration asymmetry was investigated by using nonlinear light scattering methods on lipid nanodroplets and liposomes. Nanodroplets covered with negatively charged lipids induce strong water ordering, while droplets covered with positively charged lipids induce negligible water ordering. Surprisingly, this charge-induced hydration asymmetry is reversed around liposomes. This opposite behavior in charge hydration asymmetry is caused by a delicate balance of electrostatic and hydrogen-bonding interactions. These findings highlight the importance of not only the charge state but also the specific distribution of neutral and charged lipids in cellular membranes.

The dielectric continuum model of water predicts symmetric hydration of oppositely charged ions with similar sizes. In contrast to this prediction, various phenomena indicate that positively and negatively charged ions are asymmetrically hydrated: the hydration free energies of anions are more negative compared to cations of similar size,^1−3^ the reorientation dynamics of water is different around cations and anions,^4,5^ anions preferentially adsorb to the air/water interface more than cations,^3,6,7^ and the Hofmeister series of ions that determine solubility and stability of proteins in water is more pronounced for anions compared to cations.^8,9^ A preference of water molecules toward anions indirectly emerged from these observations. A first cause for this asymmetry is the structure of water: with a more negative oxygen atom and two small more positive hydrogen atoms, it is easier for the molecule to get closer to an anion than to a cation. A second cause is the possibility for the water molecule to hydrogen (H^−^) bond with the anions but not with the cations.^3,4,10,11^

The direct test for asymmetry in the hydration of opposite charges, however, requires oppositely charged ions with identical chemical structures. A pair of large hydrophobic ions, tetraphenyl arsonium (TPA^+^) and tetraphenyl borate (TPB^–^), have been studied in this context.^12^ Earlier studies assumed that these two ions have similar hydration energies.^13,14^ However, when the water structures of TPA^+^ and TPB^–^ ions in bulk and at the surface of oil droplets dispersed in water^15^ were measured on the molecular level, it was found that TPA^+^ and TPB^–^ ions exhibit drastically different hydration behaviors both in the bulk and at the nanoscale interface. Water forms stronger and more abundant π-H bonds with TPB^–^ anions compared to the cations. This asymmetry in the hydration shell structure, along with the inherent asymmetry in the water–ion interaction and dispersion interactions, determines the charge hydration asymmetry exhibited by tetraphenyl ions.^15−17^ Furthermore, TPB^–^ ions enhance the interfacial ordering of water molecules next to oil nanodroplets, whereas TPA^+^ ions suppress the interfacial water ordering. This charge asymmetry in interfacial water ordering results from the interplay between electrostatic and H-bonding interactions. For anions both interactions are cooperative and enhance water ordering, while for cations they are anticooperative, reducing water ordering. These results clearly showed that the origin of charge hydration asymmetry cannot be explained on purely electrostatic grounds. Moreover, negative charges seem to stabilize both molecular hydration shells and macroscopic oil–water interfaces a bit more than positive charges.

The preference of water for negative ions is relevant for biological membranes, which are predominantly negatively charged.^18,19^ The anionic and cationic groups on lipid headgroups are expected to exhibit a more complex asymmetric behavior than structurally identical ion pairs, and it is of great fundamental interest to determine this behavior. Quantifying charge hydration asymmetry in the model lipid membrane requires experimental techniques that can selectively measure the interfacial water structure associated with charged lipid headgroups. Angle-resolved second harmonic scattering (SHS)^20−23^ and vibrational sum frequency scattering (SFS)^24−26^ are second-order nonlinear scattering techniques that selectively measure the response from orientationally ordered interfacial molecules at the surface of submicrometer-sized particles such as lipid droplets and liposomes.^22,27−32^ For submicrometer-sized objects dispersed in water, the nonresonant SH intensity has been shown to arise predominantly from the second-order response of oriented interfacial water molecules.^33,34^ The SHS response from water at a charged interface includes a portion of water molecules ordered via chemical interactions with the interface and another portion of molecules oriented by the electrostatic field originating from the surface. This last term reports on the surface potential.^23,35,36^ Along with interfacial water ordering, complementary information about the chemical structure and molecular ordering at the interface can be extracted from vibrational SFS. In SFS, nonresonant near-infrared photons are mixed with resonant infrared photons, which results in sum frequency photons that report on the vibrational resonances of interfacial molecules.^28−30,37^

Herein, we investigate the charge-induced hydration asymmetry of nanoscale lipid droplets and liposomes formed with oppositely charged lipids and show that the hydration asymmetry is drastically different for lipid monolayer and bilayer systems. Lipids with headgroups containing negatively charged phosphate, positively charged trimethylammonium, and zwitterionic groups were selected to mimic the realistic functional groups on biological membranes. The interfacial coverage and molecular ordering of lipid nanodroplets were measured with vibrational SFS. The negatively charged lipids form the most disordered monolayer on the oil droplet surface. However, the interfacial water ordering as measured by SHS is at a maximum for the negatively charged lipids, indicating that the electric field contributions by the lipid headgroup and the oil phase act cooperatively with each other. The two electric field components act anticooperatively for the positively charged lipids, which minimizes the net interfacial water ordering. The zwitterionic lipid droplet monolayer shows a trend between the oppositely charged lipids. The hydration asymmetry is drastically different for liposomes. SHS measurements reveal the highest interfacial water order for liposomes containing positively charged lipids, followed by negatively charged lipids, and zwitterionic liposomes order the least amount of interfacial water. The key difference between positive and negatively charged lipids is the ability of the negatively charged headgroup to H-bond with water. It turns out that this H-bonding interferes destructively with electrostatic effects arising from the charge–water interactions. The drastically different charge hydration asymmetry measured for the lipid monolayer and bilayer systems implies that the hydration asymmetry of oppositely charged ions results from a delicate balance between electrostatic and H-bonding interactions, which are further dependent on how molecules are distributed, giving extra importance to membrane geometry.

## Lipid Droplets

**Figure 1:**
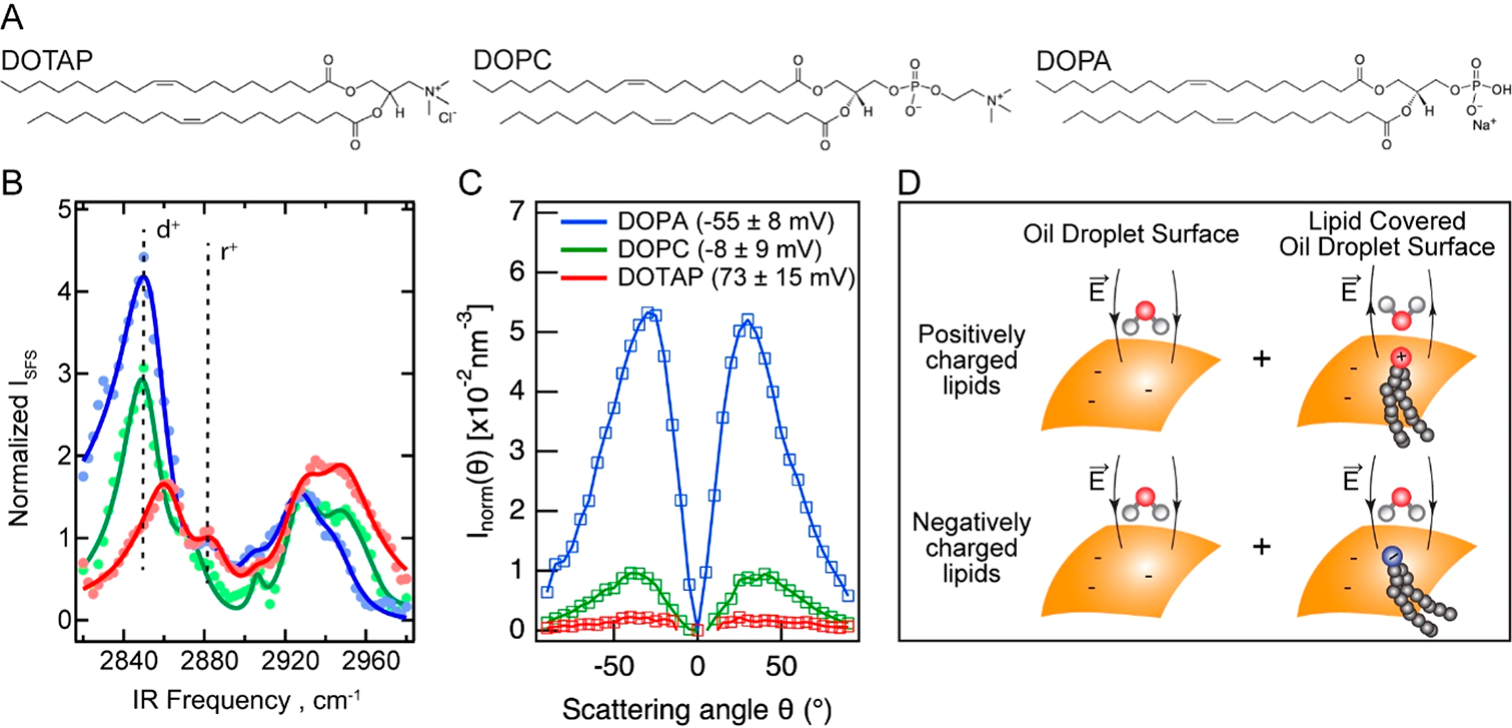
Interfacial lipid structure and hydration on lipid nanodroplets. (A) Structures of the lipids used in this study. (B) Normalized SFS spectra of DOPC (green), DOPA (blue), and DOTAP (red) nanodroplets in the C–H stretching mode region. The dashed lines indicate the positions of the d^+^ and r^+^ modes. The spectra are normalized to the intensity at 2874 cm^–1^ for comparison. Solid lines are the result of the spectral fitting detailed in the Supporting Information. The spectra are recorded with the SF and VIS beams polarized vertical (S) to the scattering plane and the IR polarized parallel (P) to the scattering plane. (C) Normalized AR-SHS patterns of oil nanodroplets with DOPC (green), DOPA (blue), and DOTAP (red) lipids, recorded using PPP polarization combination. The patterns have been corrected for intensity differences due to differences in the number density and size distribution of the nanodroplets, and the resulting scattered droplet intensity was then normalized to the intensity of bulk water measured in the SSS polarization combination. The values in parentheses next to the legend correspond to ζ-potential values. (D) Illustration of the origin of charge hydration asymmetry for DOPA and DOTAP lipid droplets. For negatively charged lipids (bottom), the electric field contributions from the oil surface and the lipid headgroups act cooperatively to enhance the interfacial water orientation. For positively charged lipids (top), the two electric field contributions act anticooperatively to suppress interfacial water ordering.

The method of ultrasonication was employed to produce droplets of *d*_34_-hexadecane that were 100 nm in size. These droplets were coated with 1 mM of lipids.^29,30,32^ Negative, positive, and zwitterionic lipid droplets were formed with 1,2-dioleoyl-*sn*-glycero-3-phosphate (DOPA), 1,2-dioleoyl-3-trimethylammonium-propane (DOTAP), and 1,2-dioleoyl-*sn*-glycero-3-phosphocholine (DOPC), respectively (Figure 1A). The interfacial water ordering of lipids with phosphate, choline, and the phosphocholine headgroups has been extensively studied in Langmuir monolayers formed at the air/water interface.^38−40^ However, water orientation by the same lipids at the oil droplet surface is expected to differ from that at the air/water interface because of the relative cooperativity between charge–charge, charge–dipole, and hydrogen-bonding interactions involving the water, oil, and the lipid molecules themselves.^29,41^ Therefore, we first characterized the molecular ordering of the three different lipid droplets using vibrational SFS.

Figure 1B shows the SFS spectra in the C–H stretching region measured using SSP (S-sum frequency, S-visible, P-infrared) polarized light, with S referring to a direction perpendicular to the scattering plane and P parallel to the scattering plane. The peaks at ∼2850, ∼2880, ∼2905, ∼2930, ∼2945, and ∼2965 cm^–1^ correspond to the symmetric (s)–CH_2_ stretch (d^+^), (s)–CH_3_ stretch (r^+^), (s)-CH_2_–Fermi resonance, antisymmetric (as)-CH_2_ stretch, (s)-CH_3_-Fermi resonance and the (as)-CH_3_ stretch, respectively.^40,42−44^ The amplitude ratio between the (s)-CH_2_ stretch and the (s)-CH_3_ stretch (d^+^/r^+^) indicates the degree of tail ordering within the monolayer. d^+^/r^+^ > 1 corresponds to a dominance of gauche defects within the lipid alkyl tails, indicating a disordered monolayer. The ratio d^+^/r^+^ ≪ 1 indicates a highly ordered monolayer with alkyl chains exhibiting an all-trans conformation.^32,45,46^ For all three lipid spectra in Figure 1B, d^+^/r^+^ > 1, indicating that all three lipids form disordered dilute monolayers at the oil droplet surface. The d^+^/r^+^ ratio is ∼1.9 for DOTAP (Figure 1B, red), ∼3.6 for DOPC (Figure 1B, green), and ∼4.0 for DOPA (Figure 1B, blue).

This high degree of disorder stems from two effects: the structure of the alkyl chains and charge–charge interactions (for net charged lipids). Starting with the first aspect, a fully saturated zwitterionic 1,2-dipalmitoyl-*sn*-glycero-3-phosphocholine (DPPC) lipid forms monolayers having almost no detectable gauche defects when identical droplets and lipid concentration are used, indicating a highly ordered monolayer.^32^ The difference between the DPPC and DOPC lipid droplet covered monolayers is related to the difference in the phase transition temperatures (*T*_m_) of both lipids: DPPC lipids are in the gel phase at room temperature, whereas the unsaturated DO lipids have a *T*_m_ well below room temperature, yielding liquid disordered monolayers at room temperature.^47,48^ For charged lipids, the second aspect of the charge–charge interaction plays an important role. Fully saturated 1,2-dipalmitoyl-*sn*-glycero-3-phosphate (DPPA) monolayers formed on lipid droplets^29^ display a similar degree of alkyl chain disorder, just like the unsaturated DOPA lipids of Figure 1B. In this case charge–charge interactions are responsible for the monolayer structure, which also happens with the formation of charged surfactant monolayers on oil droplet surfaces. As was pointed out in ref (29), due to the lack of screening across low dielectric droplets with sizes below the Debye length, the density of the monolayer is dictated by charge–charge interactions. Like charge lipids, they will be situated at larger distances compared to those in a packed monolayer to accommodate the repulsive electrostatic interactions. Furthermore, with the oil phase being intrinsically negatively charged, negatively charged amphiphiles do not penetrate into the oil, while positively charged ones do.^49^ Therefore, it is evident that the formation of charged lipid monolayers on the oil droplet surface is predominantly driven by the same interactions that drive the formation of amphiphilic surfactant monolayers. The similarity in the degree of disorder of DOPA and DPPA monolayers confirms this notion further, as the influence in chain conformation is relatively weaker than that of the electrostatic interactions.

Next, we characterize the interfacial hydration of the same lipid droplets with SHS. Figure 1C shows the angular SHS patterns of DOTAP, DOPA, and DOPC lipid covered droplets using the PPP polarization combination with all beams polarized parallel to the scattering plane. In nonresonant angle-resolved (AR)-SHS measurements, the SHS intensity reports specifically on the orientational ordering of polarizable molecules, i.e., lipid or water, along the surface normal. The SHS intensity emitted by hydrated membranes studied here mainly originates from water molecules (section S1 in the Supporting Information).^34^

Two effects modify the orientational distribution of water next to the lipid interfaces. First, the number and orientation of hydrogen bonds are perturbed by the presence of the lipid–water interface (captured by the second-order particle susceptibility, Γ^(2)^, term in eq S4 in the Supporting Information). Second, the interfacial charge causes a nonzero surface potential Φ_0_ that induces alignment of water molecules via an electrostatic charge–dipole interaction (captured by the effective third-order particle susceptibility, Γ^(3)^′, term in eq S4 in the Supporting Information).^35,36^ The length scale of water ordering by the surface electrostatic field and thus the contribution of Γ^(3)^′ to the SHS intensity depend on the ionic strength of the solution.^36^ Under physiological conditions the SH intensity reports on interfacial water <1 nm away from the interface. In the experiments demonstrated here, both Γ^(2)^, and Γ^(3)^′ are expected to contribute to the SHS intensity. From Figure 1C, it is evident that oppositely charged lipids exhibit hydration asymmetry: negatively charged DOPA lipids (Figure 1C, blue) show the maximum ordering of interfacial water, whereas the positively charged DOTAP lipids (Figure 1C, red) show the least amount of ordered water at the oil droplet surface. Interestingly, the positively charged DOTAP droplets order even less water compared to that of zwitterionic DOPC (Figure 1C, green). This is surprising, as DOPA and DOTAP droplets have comparable absolute ζ-potential values, much larger than those of DOPC droplets (see legends of Figure 1C). Therefore, one would intuitively expect the positively charged droplets to induce interfacial water ordering of the same order of magnitude as the negatively charged droplets.

To understand the origin of asymmetry next to oil droplets, we first consider the orientation of water molecules next to the bare oil droplets. Pure oil droplets carry a net negative charge on their surface,^23,50,51^ which creates an electric field that disturbs the orientation of interfacial water molecules (Figure 1D, left, bottom). The addition of negatively charged DOPA lipids adds an interfacial electric field in the same direction as that of the negatively charged oil interface, which enhances the net ordering of water molecules, giving rise to larger total nonlinear polarization and hence a larger emitted SH amplitude (Figure 1D, right, bottom). On the other hand, the electric field created by positively charged DOTAP headgroups is in the opposite direction of the electric field created by the oil surface (Figure 1D, right, top) and therefore counteracts the initial field induced water ordering. The higher degree of order exhibited by DOTAP monolayers (Figure 1B) and the near-zero SHS intensity generated by the water molecules (Figure 1C) indicate that DOTAP forms a monolayer that is ordered enough to screen the negative charge originating from the oil molecules. The high degree of disorder exhibited by DOPA (Figure 1B) points to a “patchy” monolayer in which areas of the bare oil droplet surface are exposed to water along with the lipid headgroups. In addition to the negative charge, the DOPA headgroups also carry H-bonding sites for water. Therefore, the large overall SHS intensity exhibited by the DOPA-covered droplets originates from a combination of water molecules that are oriented via electrostatic field and H-bonding together. The zwitterionic DOPC lipids generate ∼5 times lower SHS intensity compared to DOPA but a higher SHS intensity than DOTAP lipids. This intermediate trend is in accord with the observations that DOPC droplets have small yet negative ζ-potentials^30^ and PC lipids in Langmuir monolayers order water molecules with their hydrogens pointing toward the lipid headgroup, similar to negatively charged lipids.^38,39,52^ Therefore, the phosphate groups of the PC headgroups play a predominant role in ordering water molecules mostly by hydrogen bonding with water and weak electrostatic ordering effects, to a minor extent. For oppositely charged lipids, however, the cancellation of cooperativity between the different electric field contributions is sufficient to explain the vanishing orientational ordering and hydration asymmetry as seen in Figure 1D.

## Liposomes

**Figure 2:**
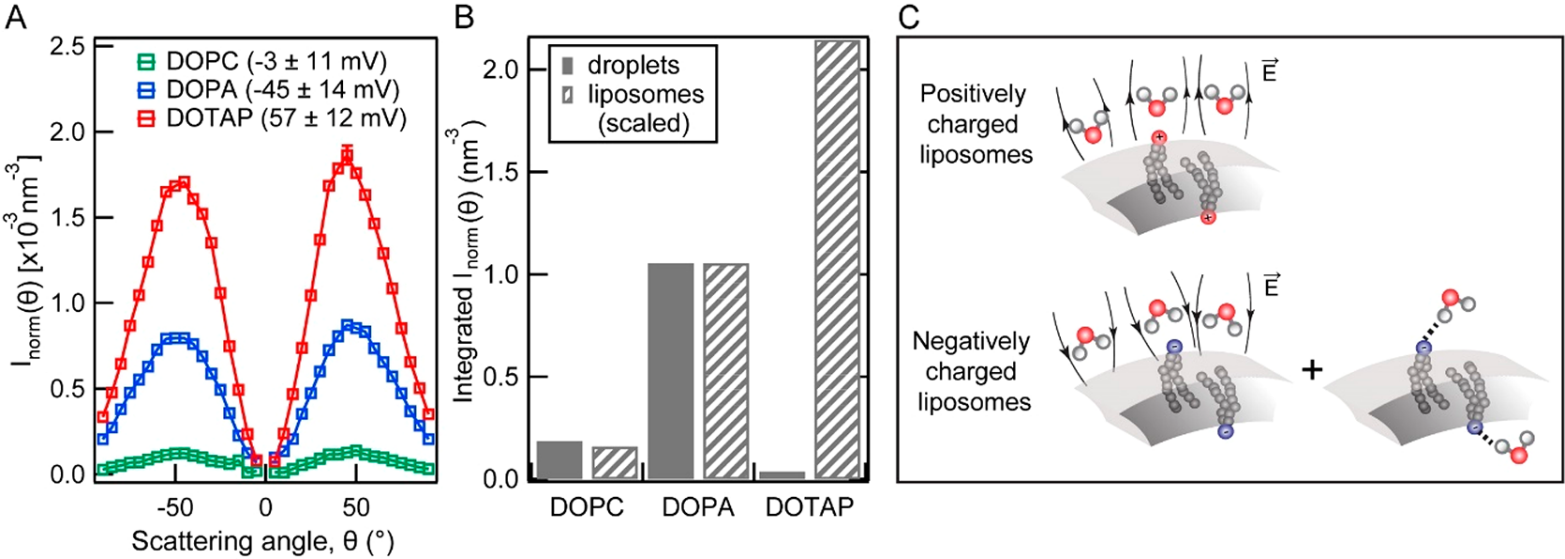
Hydration structure of liposomes. (A) Normalized AR-SHS patterns of liposomes in pure D_2_O made of DOPC (green), DOPA (blue), and DOTAP (red) lipids (0.5 mg/mL, formed by extrusion), recorded using the PPP polarization combination. The patterns have been corrected for intensity differences arising from differences in the number density and size distribution of the liposomes, and the resulting scattered intensity was then normalized to the intensity of bulk water measured by using the SSS polarization combination. The values in parentheses next to the legend correspond to ζ-potential values. (B) Comparison of the total values of *I*_norm_(θ) from the AR-SHS patterns of Figures 1C and Figure 1A, integrated from −85 to +85° scattering angles. The liposome values were scaled so that the value of DOPA liposomes matches the value of DOPA nanodroplets. (C) Illustration of the origin of interfacial hydration intensity differences for liposomes. For positively charged lipids (top), the asymmetry is dictated by the electric-field-induced ordering alone. For the negatively charged lipids, the electric-field-induced ordering (bottom left) and the hydrogen-bonding effects (bottom right) interact anticooperatively with each other.

AR-SHS patterns of 110–120 nm diameter liposomes made of DOPA (blue), DOTAP (red,) and DOPC (green) lipids are shown in Figure 2A. The intensity was corrected for the radius and the number of liposomes in the suspension, so that the intensity reflects the response of a single liposome interface as described in ref (53) and repeated in the Supporting Information for convenience. Surprisingly, in stark contrast with the result from lipid droplets, the positively charged liposomes produce the maximum SHS signal (Figure 2A, red), followed by the negatively charged liposomes (Figure 2A, blue). Zwitterionic DOPC liposomes produced the least SHS intensity (Figure 2A, green), generating ∼14 times lower SHS intensity compared with DOTAP and ∼7 times lower intensity compared with DOPA liposomes.

Figure 2B shows a comparison between the total AR-SHS intensities *I*_norm_ of droplets and liposomes integrated from −85 to +85° scattering angles. For comparison purposes, the total intensities of liposomes were scaled with the same factor so that the values of total intensities of DOPA liposomes and droplets are the same. The relative total intensities for liposomes and droplets follow a very similar trend between DOPC and DOPA membranes. Strikingly, a very big difference occurs in the DOTAP case. The SH intensity is higher for positive liposomes compared to zwitterionic and negative liposomes, while it is dramatically lower in the case of droplets. In other words, it is opposite.

In the case of liposomes, there are two lipid/water interfaces: the inner and the outer leaflets. SH photons are generated by water molecules on both sides of the membrane. The nonzero SHS intensity therefore reports on the overall transmembrane asymmetry of hydration.^37^ Negatively charged liposomes were found to have higher SHS intensity compared to zwitterionic liposomes, in agreement with previous studies,^31,37^ owing to the higher electric-field-induced water ordering. Moreover, the electric field at the center of the liposome is expected to vanish based on Gauss’ law,^54^ providing asymmetry in the electrostatic environment in the inside vs the outside of the liposome. The absence of electric field in the inside is achieved via the neutralization of the lipid headgroups by counterion pairing on the inner lipid leaflet.^55,56^

Based on electrostatic grounds alone, one would predict DOTAP and DOPA liposomes to produce similar SHS intensities, as they have ζ-potentials of roughly equal magnitude and opposite signs (see Figures 1C and 2A). Yet, clearly, DOTAP liposomes produce ∼2 times more intensity compared to DOPA and ∼14 times more intensity than DOPC liposomes. Therefore, the molecular orientational ordering of water molecules is the most asymmetric across the DOTAP membrane among all of the samples. Since the positively charged TAP group of DOTAP cannot form H-bonds with water, this group orders water molecules via the electrostatic field alone (Figure 2C, top). The asymmetry in the electric-field-induced orientation across the membrane then generates the SHS response. The asymmetry in the electric-field-induced orientation of water is expected to be similar for DOTAP and DOPA liposomes, as they carry similar charge. Then, the difference between the hydration asymmetry of DOTAP and DOPA lipids must originate from the ability of PA headgroups to hydrogen bond with water, which is absent for DOTAP. These PA interacting H-bonds are formed on both leaflets. As shown in Figure 2C (bottom panel), in addition to the electric-field-induced asymmetry, some water molecules are directly H-bonded to the negatively charged lipid headgroups, on the outside leaflet as well as on the inside leaflet. The inside leaflet’s H-bonding interaction acts anticooperatively with the electric-field-induced asymmetry on the outside and results in a lower SHS intensity for the DOPA liposomes compared to the DOTAP liposomes (Figure 2C, top panel).

The above observations highlight the need to consider the membrane chemistry in relation to the surrounding water and in terms of relevant interactions and geometry. For both liposomes and nanodroplets, the orientational ordering of water molecules occurs via a combination of (1) the electrostatic charge–dipole interaction with the charge of the lipid interface and (2) the H-bonding between lipid headgroups and neighboring water molecules. These two interactions compete or cooperate to modify the amount of water molecular ordering, resulting in different transmembrane asymmetries. The electrostatic interaction may well be symmetric with respect to the sign of charge, but the dipolar nature of the water molecule and the directionality of the H-bond induce a hydration of the charged lipid membranes that is overall asymmetric. This balance is further modified when the charges are distributed on either side of the lipid bilayer. The consequence of this balance of interactions is that the water orientational ordering around a positive lipid membrane is drastically different from that around zwitterionic or negative membranes. The water orientational ordering around positive lipid droplets is reduced depending on the lipid coverage, while it is strong and asymmetric across the membrane around positive lipid bilayers. These dramatic changes should be put into perspective with respect to the biological functions assumed by lipid asymmetry in membranes. Negatively charged lipids, even in small quantities, are distributed very specifically on different cellular lipid membranes. They play a role in the recruitment of cationic proteins or enzymes, or in phenomena like apoptosis, blood coagulation, and membrane fusion.^57,58^ On the contrary, positive lipids are very scarce, and their excess is often associated with cytotoxicity.^59,60^ These behaviors may find their origin in the subtle energy balance that comes about by balancing H-bonding, charge–dipole interactions, interfacial geometry, and system size.

## Supporting Information Available

Materials and methods, molecular sources for SHS from water around charged particles, calculation of an effective radius, and the SFS spectral fitting (PDF)
