# Supplementary material for: Liposomes and Lipid Droplets Display a Reversal of Charge-Induced Hydration Asymmetry
Source: Nano Lett. 2023 Oct 23;23(21):9858–64. doi: 10.1021/acs.nanolett.3c02653 (PMC10636888; doi:10.1021/acs.nanolett.3c02653)
Supplement: Supplementary file 1 — nl3c02653_si_001.pdf [file nl3c02653_si_001.pdf]

Supporting information for

Liposomes and Lipid Droplets Display a Reversal of Charge-Induced Hydration Asymmetry

Saranya Pullanchery[1#], Nathan Dupertuis[1#], Tereza Roesel[1], and Sylvie Roke[1,2,3*]

[1]Laboratory for Fundamental BioPhotonics (LBP), Institute of Bioengineering (IBI), School of Engineering (STI) École Polytechnique Fédérale de Lausanne (EPFL), CH-1015 Lausanne, Switzerland
[2]Institute of Materials Science (IMX), École Polytechnique Fédérale de Lausanne (EPFL), CH-1015 Lausanne, Switzerland
[3]Lausanne Centre for Ultrafast Science (LACUS), École Polytechnique Fédérale de Lausanne (EPFL), CH-1015 Lausanne, Switzerland,
#both authors contributed equally
e-mail*: sylvie.roke@epfl.ch

Materials and methods

Chemicals. Sulfuric acid (95-97%, ISO, Merck), ammonium hydroxide (30%, Sigma-Aldrich), hydrogen peroxide (30%, Reactolab SA), chloroform (Emsure, ACS, ISO, Merck), cholesterol, phosphorus standard solution (0.6 M, Sigma-Aldrich), L-ascorbic acid (ACS, ≥99%, Sigma-Aldrich), ammonium molybdate (VI, ACS, 81-83%, Sigma-Aldrich), and d_{34}-hexadecane ($C_{16}D_{34}$, 98% d, Cambridge Isotope) were used as received. 1,2-dioleoyl-*sn*-glycero-3-phosphocholine (DOPC) and 1,2-dioleoyl-*sn*-glycero-3-phospho-L-serine (DOPS, sodium salt), 1,2-dipalmitoyl-sn-glycero-3-phosphocholine (DPPC), and 1,2-dipalmitoyl-sn-glycero-3-phospho-L-serine (DPPS, sodium salt) were purchased in powder form (>99%) from Avanti Polar Lipids (Alabama, USA) and stored at -20 °C until further use. Ultrapure H_2O was obtained with a Milli-Q UF plus instrument (Millipore, Inc.), with an electrical resistance of 18.2 MΩ cm. D_2O used was 99.8% D atoms with an electrical resistance of >2 MΩ cm (Armar). The chemical structures of the used lipids are presented in Fig.1A.

Cleaning procedures. Glassware for liposomes preparation was cleaned with a Deconex® (Borer Chemie AG) solution prepared by 1:20 dilution with ultrapure water (Milli Q, Millipore, Inc., electrical resistance of 18.2 MΩ cm), then rinsed thoroughly with ultrapure water at least 15 times. Glassware for the phosphate assay required a two-step cleaning procedure: First a cleaning with a 3:1 H_2SO_4:H_2O_2 solution was done, which was followed by a cleaning with a 3:1 NH_4OH_4:H_2O_2 solution at 100 °C, each for 10 minutes. After and in between the cleaning steps the glassware was thoroughly rinsed with ultrapure water.

Sample preparation. Oil nanodroplets with a lipid monolayer were prepared according to the procedure described in Ref.[1], with 2 vol % d_{34}-hexadecane in D_2O. We mixed the solutions with lipid powders to reach a lipid concentration of 1 mM at a temperature of at least 5 °C above the transition temperature of the phospholipid by using a hand-held homogenizer (TH, OMNI International) for 4 min and an ultrasonic bath (35 kHz, 400 W, Bandelin) for the same duration. The resultant droplet system was used for SFS measurements and was diluted to 0.1 vol % hexadecane with D_2O for SHS measurements. The size distribution of the nanodroplets was measured with dynamic light scattering (DLS, Zetasizer Nano ZS, Malvern). The nanodroplets had a mean hydrodynamic radius in the range of 170-200 nm with a polydispersity index (PDI) of less than 0.25. The hydrodynamic radii were calculated from the intensity autocorrelation function using the optical properties of the liquids (d_{34}-hexadecane and D_2O). The samples were stored and measured in sealed cuvettes. All measurements were performed at 24 °C.

Small unilamellar vesicles (liposomes) were prepared by extrusion according to Ref.[2-4]. To create multilamellar vesicles, typically 10 mg of lipid powder was dissolved in chloroform in a glass vial. Subsequently, the chloroform was evaporated with a nitrogen gas stream while rotating the vial. The resulting lipid film was further dried in vacuum (<100 mbar, created by an oil-free diaphragm pump) at room temperature for at least 2 hours. Finally, the lipid film was re-suspended in D_2O and vortexed. To create unilamellar vesicles, the resulting multilamellar vesicle solutions were extruded with a Miniextruder (Avanti Polar Lipids) using a polycarbonate membrane (Avanti Polar Lipids) with pore diameter of 200 nm at room temperature. Unilamellar vesicles were stored in closed containers up to 4 weeks at 4 °C. The size and ζ-potential distribution of the liposomes were also measured with DLS and electrophoretic measurements at 23 °C (Malvern ZS nanosizer). To determine the size distribution of the vesicles, three subsequent measurements, each 11 runs, were averaged. To determine the ζ-potential of the vesicles three subsequent measurements, each 30 runs at automated voltage, were averaged. The liposomes were found to have a mean diameter in the range of 120-145 nm with a polydispersity index (PDI) of less than 0.2. The final lipid concentration was determined using a calorimetric phosphorus assay; for further details see Ref.[5] and the protocol at Ref.[6]. The concentration of the lipids in the sample was 0.5 mg lipids/ml weight ratio for DLS, ζ-potential measurements, and SHS experiments.

Angle-resolved second harmonic scattering. The light source for AR-SHS measurements was a Yb:KGW laser (Pharos SP, Light Conversion) producing 190 fs pulses centered at 1028 nm with a 200 kHz repetition rate. The polarization of the incident pulses was controlled via a Glan-Taylor polarizer (GT10-B, Thorlabs) and a zero-order half-wave plate (WPH05M-1030, Thorlabs). The incident laser pulses were filtered via a long-pass filter with a cut-off wavelength at 750 nm (FEL0750, Thorlabs). The average incident power at the sample was 60 mW (0.3 µJ per pulse). The laser beam was focused into a disposable cylindrical glass cuvette with an inner diameter of 4.2 mm (LS instruments). The beam waist in the sample was 35 µm at the focus with a Rayleigh length of 0.94 mm. SHS light at 514 nm was collected with a plano-convex lens (f = 50 mm) and then filtered through a 10 nm-wide bandpass filter centered at 516 nm (ET516/10, Chroma). A Glan-Taylor polarizer (GT10-A, Thorlabs) was used for the polarization analysis of the scattered light. The polarization of the incident and outgoing light was either parallel (P) or perpendicular (S) with respect to the horizontal scattering plane. The SH light was focused using a plano-convex lens (f = 25 mm) into a gated photo-multiplier tube (PMT, H7421-40, Hamamatsu). A more detailed description of the setup can be found in Ref.[67]

Scattering patterns were recorded in 5° steps between -85° and +85° (0° corresponding to the forward direction of the fundamental beam) with an opening angle of 3.4°. The acceptance

angle was controlled by an iris and set to 3.4°. The intensity was measured in PPP polarization combination, then normalized to the intensity of neat water in PPP (to remove the contribution from the bulk) and in SSS polarization combination (to correct for variations between different data sets).

$$S(\theta) = \frac{I(\theta)_{PPP,sample} - I(\theta)_{PPP,water}}{I(\theta)_{SSS,water}} \qquad \text{(S1)}$$

where PPP and SSS refer to the polarization combination of the incident and outgoing light (the first letter refers to the polarization state of the SH beam and the second and third letter refer to that of the fundamental beam; P (S) is parallel (perpendicular) to the scattering plane). The integrated fs-ESHS intensity was calculated by summing the normalized fs-ESHS intensity over all angles between -85° and 85° (except for 0°) using the formula:

$$S_{int} = \sum_{\theta \neq 0} S(\theta) \qquad \text{(S2)}$$

Normalization for AR-SHS. Intensity was scaled with respect to radii and number of nanodroplets/liposomes computed from the size distribution and sample preparation. This allows qualitative comparison of patterns between different samples.

The size independent SHS response per nanodroplets or liposome, I_{norm}, was computed by taking into account the number N_p of liposomes in the solution and their radius R:

$$\alpha(\theta) = \frac{S(\theta)}{N_p R^6} = I_{norm}(\theta, R) \qquad \text{(S3)}$$

In addition, the polydispersity of the samples was corrected by the computation of an effective radius R_{eff} (see section S2).

Sum Frequency Scattering measurements. The experimental set-up for vibrational sum frequency scattering measurements has been described previously in detail[7-8]. An infrared (IR) and a visible (vis) laser beams were spatially and temporally overlapped at an angle of 15° in a sample cell with a path length of 200 μm. The IR pulse was centered at 2900 cm^{-1} (FWHM = 160 cm^{-1}) and the vis pulses at 12500 cm^{-1} (FWHM = 15 cm^{-1}) at a repetition rate of 1 kHz. The SFS light was measured at a scattering angle of 55°, collected and collimated by a plano-convex lens (f = 15mm, Thorlabs LA1540-B). The polarization of the IR beam was controlled by two BaF_2 wire grid polarizers (Thorlabs, WP25H-B), the vis beam by a polarizing beam splitter cube (CVI, PBS-800-050) and a half-wave plate (EKSMA, 460-4215 and the SF beam by a Glan−Taylor prism (Thorlabs, GT15-B). The SF beam was filtered by two short wave pass filters (3rd Millenium, 3RD770SP) before being spectrally dispersed with a monochromator (Acton, SpectraPro 2300i) and detected with an intensified CCD camera (Princeton Instruments, PI-Max3). The gate width was set to 10 ns, and the acquisition time for a single spectrum was 600 s. All SFS spectra were normalized by the infrared profile, measured as the

sum frequency signal from a solid film of 100 nm $BaTiO_3$ particles detected under the same experimental geometry.

S1. Origin of the SHS response from hydrated lipid membranes

Under illumination by an electric field $\widetilde{\boldsymbol{E}}(\omega)$, the second-order polarization $\boldsymbol{P}^{(2)}$ (i.e., the average second-order response) of the medium is composed by the sum of the induced molecular polarizations $\boldsymbol{p}^{(2)}$ per unit of volume[9]. This is expressed as: $\boldsymbol{P}^{(2)}(2\omega) = \varepsilon_0 \boldsymbol{\chi}^{(2)}: \widetilde{\boldsymbol{E}}(\omega)\widetilde{\boldsymbol{E}}(\omega) = N\langle \boldsymbol{p}^{(2)} \rangle$, where $\boldsymbol{\chi}^{(2)}$ is the second-order susceptibility tensor that is characteristic of the material, ε_0 is the vacuum permittivity, N is the number density of probed molecules. Under our measurement conditions, water and lipids molecules are non-resonant. Therefore, using the anharmonic oscillator model[9], we can assume that the $\chi^{(2)}$ value is about the same order of magnitude for a lipid and a water molecule. This means that individual lipid and water molecules generate about the same amount of second harmonic light. As a result, the respective contributions to the total SH intensity from these molecules at the hydrated membrane surface depends on their number densities. In a volume defined by a section of 10 nm^2 of the lipid interface and extending up to 3 nm from the lipid layer, there are $d^{-1} \times 10\, nm^2 \times 3\, nm \approx 1000$ water molecules (where $d = 33.2\, nm^{-3}$ is the number density of heavy water in ambient conditions). Considering the same 10 nm^2 section of the interface, and an average lipid headgroup area of ~0.66 nm^2, 15 lipid molecules are in contact with water. As the SH intensity scales with the square of the number density of scattering molecules, this means that the ratio of intensity from water molecules over intensity from lipid molecules is around $\left(\frac{1000}{15}\right)^2 \sim 4444$. Therefore, SHS intensity emitted by hydrated membranes is originating mainly from water molecules, as shown by Yan et al[10].

The molecular sources for SHS from water around charged particles

There are two types of interactions contributing to the ordering (i.e. alignment of water along the surface normal) of water molecules and thus to the SHS intensity. First, the presence of charges at the surface of the particle generates an electrostatic field. The electrostatic potential decays exponentially as $\Phi(r) = \Phi_0 \frac{R}{r} e^{-\kappa(r-R)}$, where R is the radius of the particle, r is the radial distance to the particle center, κ is the Debye length, and Φ_0 is the surface potential of the particle. This can induce a third-order response by two pathways[11]: 1) the incident light interacts with this continuous electrostatic field by generating a purely third-order response from water molecules; 2) the DC field induces a symmetry-breaking reorientation of water molecules both at the interface and further in the so-called diffuse double layer, this reorientation then resulting in a second-order process. Since the second-order responses in both pathways depend linearly on the incident electric fields and on the electrostatic field, we

group them under an effective third-order susceptibility tensor $\chi^{(3)\prime}$ as described in Ref.[11]. Second, the orientational order induced by any other chemical interaction (e.g. hydrogen bonding) at the surface can be represented by the surface second-order susceptibility tensor $\chi_S^{(2)}$. These two sources are represented respectively by the effective third-order and effective second-order particle surface susceptibility $\Gamma^{(2)}$ and $\Gamma^{(3)\prime}$ that depend on R, $\chi_S^{(2)}$, $\chi^{(3)\prime}$, and the scattering angle θ. These effective susceptibility quantities capture the scattering geometry of the experiment, the interfacial structure, and the electrostatic field altogether. They are defined in more detail in Refs.[11-13] The total SHS intensity coming from the particles in solution is therefore proportional to:

$$I_{2\omega} \propto \left|\Gamma^{(2)}\left(R, \chi_S^{(2)}, \theta\right) + \Gamma^{(3)\prime}\left(R, \chi^{(3)\prime}, \theta\right)\Phi_0\right|^2 \quad \text{(S4)}$$

S2. Calculation of an effective radius

To obtain the intensity for a single liposome, we normalized the measured values of $S(\theta)$ of Eq. (S1) by the number of liposomes (N_{lip}), obtained from the amount of lipid used and the DLS distribution. We also correct for the difference in the radius (R) in the following way. For a monodisperse solution with N_{lip} liposomes that are smaller than ~250 nm in radius and that each scatter an intensity $I(\theta)$, the total scattered signal $S(\theta)$ scales as follows[14]:

$$S(\theta) = I(\theta)N_{lip} \propto \alpha(\theta)N_{lip} \quad \text{(S5)}$$

The factor α contains all the information about the SHS response per liposome, independent of its size. Thus, to compare the water response per liposome with radius R, we use:

$$\alpha(\theta) = \frac{S(\theta)}{N_{lip}R^6} \quad \text{(S6)}$$

For the SHS experiments we correct for polydispersity by replacing the radius R in Eq. (S6) with an effective radius (R_{eff}). Since all particles contribute in the same way to the overall intensity of any light scattering experiment (linear or nonlinear), we can use linear light scattering data obtained from dynamic light scattering (DLS) to compute this effective radius R_{eff} that can be used for the SHS data normalization. Thus, we will use the DLS size distribution to determine a single effective radius that produces the same scattered intensity as the measured DLS size distribution. In other words, we replace the total DLS intensity from a polydisperse distribution $\sum_i I(R_i)$ by an intensity $\alpha(\theta, R_{eff})$ from a 'monodisperse' solution. The obtained effective radius is then used to normalize the SH intensity according to Eq. (S6).

In this way, we exclude intensity differences based on different sample sizes / size distributions and obtain the nonlinear scattering response of a single liposome $\alpha(\theta)$, which contains information about the change in the orientational distribution of water between the inner and outer leaflets of the liposome.

DLS uses the temporal autocorrelation of scattered light to measure an intensity-weighted particle size distribution histogram. The output of such a measurement is a normalized distribution $D(R)$, which we will use here to correct the SHS signal for variations in the liposome size distribution. Explicitly we have from Eq. (S6):

$$\alpha(\theta) = \frac{\sum_i I_i(\theta)}{N_{lip}R^6} = \frac{S(\theta)}{N_{lip}{R_{eff}}^6} \tag{S7}$$

In the Rayleigh Gans Debye limit, which is applicable here[15], the intensity of scattered light in a DLS measurement also scales with R^6, so that:

$$D(R) = \frac{P(R)R^6}{\int P(R)R^6 dR} \tag{S8}$$

The particle size distribution $P(R)$ is a normalized probability distribution, such that $\int P(R)dR = 1$. The particle size distribution can be found from the DLS intensity-weighted distribution by:

$$P(R) = \frac{D(R)/R^6}{\int \frac{D(R)}{R^6} dR} \tag{S9}$$

Using the particle size distribution, we then calculate the effective radius for the liposomes using the following general expression as described in Ref.[16] (SI, S2-S3):

$$R_{eff,lip} = \left[\frac{\int P(R)R^6 dR}{\int \frac{1}{2} P(R)(R^2+(R-d)^2)dR}\right]^{\frac{1}{4}} \tag{S10}$$

where the denominator takes into account that with changing radius the number of lipids per liposomes changes too (i.e. size and number density are related). For nanodroplets, we have the following expression:

$$R_{eff,d} = \left[\frac{\int P(R)R^6 dR}{\int \frac{1}{2} P(R)R^3 dR}\right]^{\frac{1}{3}} \tag{S11}$$

Where the denominator is now representing a sphere rather than a hollow shell.

S3. Fitting of SFS measurements

The SFS spectra were fitted using the equation:

$$I_{SF}(\omega_{IR}) \propto \left| A_{NR} e^{i\phi_{NR}} + \sum_\nu \frac{A_\nu \gamma_\nu}{\omega_{IR} - \omega_\nu + i\gamma_\nu} \right|^2 \tag{S12}$$

where A_{NR} and ϕ_{NR} are the amplitude and phase of the nonresonant background, A_v, ω_v, and γ_v denote the amplitude, resonant frequency, and linewidth of the resonant vibrational modes. ω_{IR} is the frequency of the infrared beam. The spectra were fit using Levenberg-Marquadt iterations in Igor Pro.

Table S1. Parameters used to fit the data in Fig. 1B

	DOPC			DOPA			DOTAP		
	ω_v (cm^{-1})	γ_v(cm^{-1})	A_v	ω_v(cm^{-1})	γ_v(cm^{-1})	A_v	ω_v(cm^{-1})	γ_v(cm^{-1})	A_v
d^+	2851	12	16	2851	14	20.8	2863	14	9.3
r^+	2874	10	4.4	2878	12	5.2	2884	12	4.8
d^{+FR}	2906	8	1.1	2906	9	2.0	2906	8	1.0
d^-	2927	12	10.1	2928	14	11.4	2928	16	10.5
r^{+FR}	2945	12	5.1	2945	14	8.0	2947	16	9.0
r^-	2965	12	-3.0	2960	14	-0.1	2960	14	-0.1
A_{NR}	0.1			-1.7			0.1		
ϕ_{NR}	-40°			-11°			-40°		

References

1. Chen, Y.; Jena, K. C.; Lütgebaucks, C.; Okur, H. I.; Roke, S. Three Dimensional Nano "Langmuir Trough" for Lipid Studies. *Nano Lett.* **2015,** *15*, 5558-5563.
2. Hope, M. J.; Bally, M. B.; Webb, G.; Cullis, P. R. Production of Large Unilamellar Vesicles by a Rapid Extrusion Procedure. Characterization of Size Distribution, Trapped Volume and Ability to Maintain a Membrane Potential. *Biochim. Biophys. Acta - Biomembr.* **1985,** *812*, 55-65.
3. MacDonald, R. C.; MacDonald, R. I.; Menco, B. P. M.; Takeshita, K.; Subbarao, N. K.; Hu, L.-r. Small-Volume Extrusion Apparatus for Preparation of Large, Unilamellar Vesicles. *Biochim. Biophys. Acta - Biomembr.* **1991,** *1061*, 297-303.
4. Mayer, L. D.; Hope, M. J.; Cullis, P. R. Vesicles of Variable Sizes Produced by a Rapid Extrusion Procedure. *Biochim. Biophys. Acta - Biomembr.* **1986,** *858*, 161-168.
5. Chen, P. S.; Toribara, T. Y.; Warner, H. Microdetermination of Phosphorus. *Anal. Chem.* **1956,** *28*, 1756-1758.
6. Avanti Polar Lipids. https://avantilipids.com/ (accessed 1/5/2019).
7. de Aguiar, H. B.; Samson, J.-S.; Roke, S. Probing Nanoscopic Droplet Interfaces in Aqueous Solution with Vibrational Sum-Frequency Scattering: A Study of the Effects of Path Length, Droplet Density and Pulse Energy. *Chem. Phys. Lett.* **2011,** *512*, 76-80.
8. de Aguiar, H. B.; Scheu, R.; Jena, K. C.; de Beer, A. G. F.; Roke, S. Comparison of Scattering and Reflection Sfg: A Question of Phase-Matching. *Phys. Chem. Chem. Phys.* **2012,** *14*, 6826-6832.
9. Boyd, R. W., *Nonlinear Optics (Third Edition)*. Academic Press: Burlington, 2008.
10. Yan, E. C. Y.; Liu, Y.; Eisenthal, K. B. New Method for Determination of Surface Potential of Microscopic Particles by Second Harmonic Generation. *J. Phys. Chem. B* **1998,** *102*, 6331-6336.
11. de Beer, A. G. F.; Campen, R. K.; Roke, S. Separating Surface Structure and Surface Charge with Second-Harmonic and Sum-Frequency Scattering. *Phys. Rev. B* **2010,** *82*, 235431.

12. Lütgebaucks, C.; Gonella, G.; Roke, S. Optical Label-Free and Model-Free Probe of the Surface Potential of Nanoscale and Microscopic Objects in Aqueous Solution. *Phys. Rev. B* **2016,** *94*, 195410.
13. Roke, S.; Bonn, M.; Petukhov, A. V. Nonlinear Optical Scattering: The Concept of Effective Susceptibility. *Phys. Rev. B* **2004,** *70*, 115106.
14. de Beer, A. G. F.; Roke, S. Nonlinear Mie Theory for Second-Harmonic and Sum-Frequency Scattering. *Phys. Rev. B* **2009,** *79*, 155420.
15. de Beer, A. G. F.; Roke, S. Sum Frequency Generation Scattering from the Interface of an Isotropic Particle: Geometrical and Chiral Effects. *Phys. Rev. B* **2007,** *75*, 245438.
16. Smolentsev, N.; Lütgebaucks, C.; Okur, H. I.; de Beer, A. G. F.; Roke, S. Intermolecular Headgroup Interaction and Hydration as Driving Forces for Lipid Transmembrane Asymmetry. *J. Am. Chem. Soc.* **2016,** *138*, 4053-4060.
